# Chiari malformation and central sleep apnea syndrome: efficacy of treatment with adaptive servo-ventilation*

**DOI:** 10.1590/S1806-37132014000500014

**Published:** 2014

**Authors:** Jorge Marques do Vale, Eloísa Silva, Isabel Gil Pereira, Catarina Marques, Amparo Sanchez-Serrano, António Simões Torres

**Affiliations:** Tondela-Viseu Hospital Center, Viseu, Portugal; Tondela-Viseu Hospital Center, Viseu, Portugal; Tondela-Viseu Hospital Center, Viseu, Portugal; Tondela-Viseu Hospital Center, Viseu, Portugal; Tondela-Viseu Hospital Center, Viseu, Portugal; Tondela-Viseu Hospital Center, Viseu, Portugal

**Keywords:** Sleep apnea, central, Arnold-Chiari malformation, Noninvasive ventilation

## Abstract

The Chiari malformation type I (CM-I) has been associated with sleep-disordered breathing, especially central sleep apnea syndrome. We report the case of a 44-year-old female with CM-I who was referred to our sleep laboratory for suspected sleep apnea. The patient had undergone decompressive surgery 3 years prior. An arterial blood gas analysis showed hypercapnia. Polysomnography showed a respiratory disturbance index of 108 events/h, and all were central apnea events. Treatment with adaptive servo-ventilation was initiated, and central apnea was resolved. This report demonstrates the efficacy of servo-ventilation in the treatment of central sleep apnea syndrome associated with alveolar hypoventilation in a CM-I patient with a history of decompressive surgery.

## Introduction

The Chiari malformation type I (CM-I) is characterized by caudal displacement of the cerebellum and by herniation of the cerebellar tonsils through the foramen magnum.^(^ 1 ^)^ This malformation has been associated with sleep-disordered breathing, especially central sleep apnea syndrome.^(^ 2 ^)^ Treatment of symptomatic CM-I consists of surgical decompression, which usually resolves the associated sleep-disordered breathing.^(^ 3 ^)^ We report a case of severe central sleep apnea syndrome in a female CM-I patient with a history of decompressive surgery who was treated effectively with adaptive servo-ventilation (ASV).

## Case report

**Figure 1:**
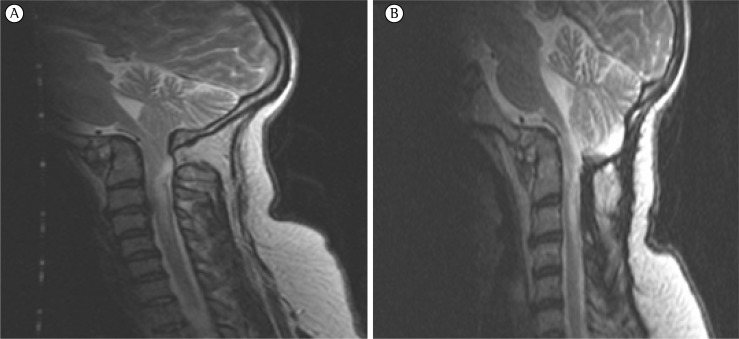
In A, presurgical magnetic resonance imaging scan of the brain showing bony malformation of the craniovertebral junction associated with basilar impression and a shortened clivus. Low cerebellar tonsils (Chiari malformation type I), but without evidence of syringomyelia. In B, postsurgical magnetic resonance imaging scan of the brain (T2) showing bony malformation of the craniovertebral junction associated with syringomyelia at the level of C2 and C3.

A 44-year-old female patient diagnosed with CM-I in 2008 (Figure 1A) underwent decompressive surgery of the posterior cranial fossa. She underwent suboccipital craniectomy, laminectomy of C1 and C2, and duraplasty. Her postoperative complications included cerebrospinal fluid fistula, which required reoperation and application of biological glue, and surgical wound infection, which was treated with debridement. Twelve months after surgery, the patient presented with worsening of neurological symptoms, including occipital headaches, dizziness, gait imbalance, left facial paresis, and dysphagia for liquids. Magnetic resonance imaging showed bony malformation of the craniovertebral junction associated with a syringomyelic cavity involving C2 and C3 (Figure 1B). Additional surgical intervention was ruled out because of the associated risk of respiratory depression. Three years after surgery, the patient was referred to our sleep laboratory for suspected sleep apnea. She reported nonrestorative sleep and morning headaches, but she had no daytime sleepiness (Epworth score of 4) or symptoms suggestive of restless legs syndrome or narcolepsy. Additional information obtained from the patient's family confirmed the presence of snoring and witnessed apneas. The patient had a history of hypothyroidism and received levothyroxine regularly. She was a nonsmoker and did not drink alcohol.

Physical examination revealed that the patient had a body mass index of 34 kg/m^2^, a systemic blood pressure of 127/73 mmHg, and a neck circumference of 46 cm. In addition, she had a hypertrophic soft palate (Mallampati class II), but she had no facial dysmorphisms. The remainder of the physical examination was normal.

**Figure 2:**
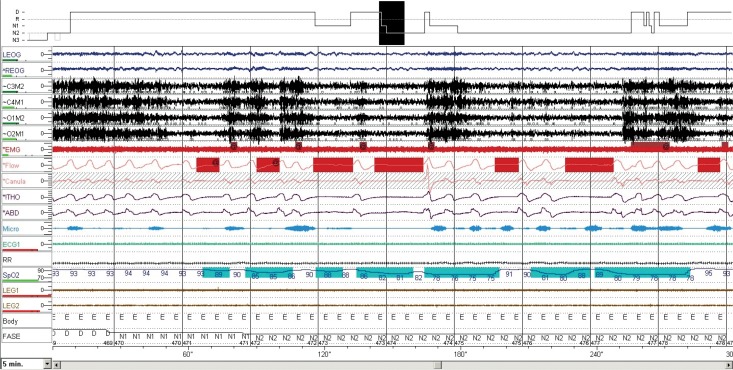
Baseline polysomnogram showing central apneas.

A chest X-ray was unremarkable. There was no evidence of cardiovascular comorbidities (an echocardiogram and a Holter examination were unremarkable). Thyroid function was normal. An arterial blood gas analysis showed severe hypoxemia with mild hypercapnia (FiO_2_ = 0.21; pH = 7.35; PaO_2_ = 51 mmHg; PaCO_2_ = 56 mmHg; and SaO_2_ = 89%). Respiratory function test results revealed a slight reduction in FVC, a reduction in expiratory reserve volume and a preserved TLC. Polysomnography showed low sleep efficiency (44.4%), with 333 central respiratory events, a respiratory disturbance index of 108 events/h, and 27.6% of sleep time with oxyhemoglobin saturation < 90% (Figure 2).

**Figure 3:**
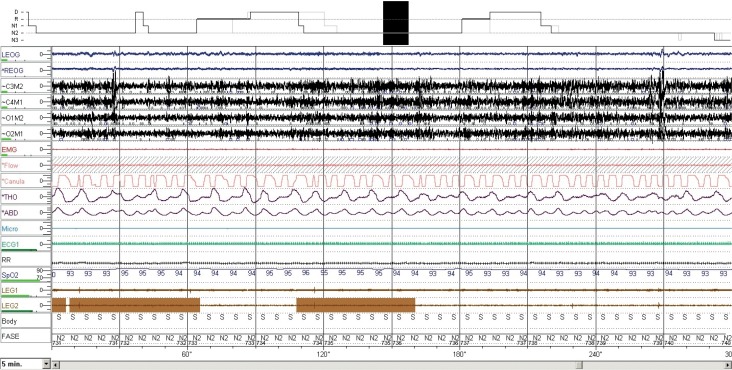
Polysomnogram under servo-ventilation, showing resolution of the central apneas.

The patient started treatment for central sleep apnea syndrome with ASV (S9 Autoset CS(tm); ResMed Corp., San Diego, CA, USA), with a maximum pressure support of 15 cmH_2_O, a minimum pressure support of 5 cmH_2_O, an expiratory pressure of 8 cmH_2_O, and an RR of 15 breaths/min. After six months of treatment, polysomnography under ASV showed that the respiratory disturbance index improved from 108 events/h to 4.8 events/h and that the patient spent 1.4% of sleep time with oxyhemoglobin saturation < 90% (Figure 3). In addition, there was improvement in gas exchange (FiO_2_ = 0.21; pH = 7.36; PaO_2_ = 69 mmHg; PaCO_2_ = 46 mmHg; and SaO_2_ = 93%).

## Discussion

The CM-I has been defined as > 5-mm caudal displacement of the cerebellar tonsils through the foramen magnum and is usually associated with a volumetrically reduced posterior fossa.^(^ 1 ^)^ For a diagnosis, these radiological criteria should be interpreted in the clinical context, and magnetic resonance imaging is the most useful imaging tool. The respiratory center that controls breathing is located in the medulla oblongata, at the level of the craniocervical junction, and can be affected in this disease, thereby leading to respiratory disorders, especially during sleep. ^(^ 4 ^)^ Studies in the literature have reported a high prevalence of sleep-disordered breathing in patients with CM.^(^ 5 ^,^ 6 ^)^ Although most reports in adults describe especially central sleep apneas, mixed and obstructive apneas are also seen.^(^ 7 ^,^ 8 ^)^ Central apneas might result from direct compression of the central respiratory components, compression of cranial nerve pairs IX and X, and afferent nerve lesion caused by syringomyelic cavities.^(^ 9 ^)^ Hypoventilation is defined as sustained oxygen desaturation that is not associated with obstructive apneas, hypopneas, or periodic breathing.^(^ 10 ^)^ Patients with daytime hypercapnia, mainly because of neuromuscular disease or ventilatory control abnormalities (obesity hypoventilation syndrome and central alveolar hypoventilation), may also have central apneas during sleep.^(^ 11 ^)^ Central events are characterized by a temporary cessation of the neural respiratory drive during sleep, resulting in a decrease in ventilation and changes in gas exchange.^(^ 12 ^)^ In general, central apneas during sleep in patients with hypercapnia should be distinguished from those occurring in patients with normocapnia or hypocapnia. Hypercapnic central sleep apnea overlaps with hypoventilation syndromes and is considered an integral part of sleep hypoventilation syndrome.^(^ 11 ^)^

In the case described here, there were changes in respiratory function, including a slight reduction in FVC accompanied by a reduction in expiratory reserve volume. In obese patients, there is respiratory mechanics impairment that causes changes in pulmonary function, such as increased work of breathing and reduced lung volumes. The ventilatory restriction imposed by obesity is usually mild and is attributed to the mechanical effects that accumulation of adipose tissue has on the diaphragm and chest wall: diaphragmatic excursion is impaired and chest compliance is decreased.^(^ 13 ^)^ The reduction in expiratory reserve volume may be detectable even in modestly overweight patients. In patients with morbid obesity, this change may be accompanied by a reduction in TLC and functional residual capacity.^(^ 14 ^)^ Some obese patients have alveolar hypoventilation. The mechanism through which obesity leads to hypoventilation is complex and has yet to be fully understood. Several mechanisms have been proposed, including changes in respiratory mechanics, decreased central responses to hypercapnia and hypoxia, and neurohormonal changes, such as resistance to leptin.^(^ 15 ^)^ Obesity hypoventilation syndrome is defined as a combination of obesity (body mass index ≥ 30 kg/m^2)^, daytime hypercapnia, and different types of sleep-disordered breathing in the absence of other conditions that may cause alveolar hypoventilation (obstructive or restrictive lung diseases, diseases of the chest wall, and neuromuscular diseases).^(^ 16 ^)^ Patients with neurological disorders, including CM, may have central hypoventilation.^(^ 17 ^)^ However, alveolar hypoventilation associated with central apneas is not common in CM-I, and, in the present case, it is not possible to exclude the role of obesity in the changes found on arterial blood gas analysis.

Decompressive surgery usually results in a decreased number of respiratory events during sleep and reduces sleep fragmentation in a significant number of patients, with the effects being more pronounced in those with central apneas.^(^ 3 ^)^ However, there are reports of the emergence of central apneas after surgery.^(^ 18 ^)^

ASV is a form of closed-loop mechanical ventilation, pressure preset, and volume or flow cycled. It alleviates central apneas by providing dynamic (breath-by-breath) adjustment of inspiratory pressure support with a back-up rate to normalize breathing patterns. The efficacy of ASV has been established especially in the treatment of central sleep apnea syndrome associated with congestive heart failure. In central sleep apnea syndrome associated with neurological disorders (without Cheyne-Stokes respiration), the role of ASV has yet to be well established.^(^ 19 ^)^

The clinical case reported here demonstrates the efficacy of ASV in the treatment of central sleep apnea syndrome associated with alveolar hypoventilation in a CM-I patient, since there was complete resolution of the central events and a significant improvement in gas exchange. In addition, the case suggests that ASV may be efficacious in the treatment of central sleep apnea in CM-I patients with a history of decompressive surgery. We have found only one similar case reported in the published literature. ^(^ 20 ^)^ In conclusion, ASV may be an alternative to decompressive surgery in the treatment of central sleep apnea in CM-I patients
