# Supplementary material for: Chiari malformation and central sleep apnea syndrome: efficacy of treatment with adaptive servo-ventilation
Source: J Bras Pneumol. 2014 Sep-Oct;40(5):574–8. doi: 10.1590/S1806-37132014000500014 (PMC4263339; doi:10.1590/S1806-37132014000500014)
Supplement: Translation

# Translation: Malformação de Chiari e síndrome de apneia central do sono: eficácia do tratamento com servoventilação adaptativa*

**Authors:** Jorge Marques do Vale, Eloísa Silva, Isabel Gil Pereira, Catarina Marques, Amparo Sanchez-Serrano, António Simões Torres

**Affiliations:** Centro Hospitalar Tondela-Viseu, Viseu, Portugal; Centro Hospitalar Tondela-Viseu, Viseu, Portugal; Centro Hospitalar Tondela-Viseu, Viseu, Portugal; Centro Hospitalar Tondela-Viseu, Viseu, Portugal; Centro Hospitalar Tondela-Viseu, Viseu, Portugal; Centro Hospitalar Tondela-Viseu, Viseu, Portugal

**Keywords:** Apneia do sono tipo central, Malformação de Arnold-Chiari, Ventilação não invasiva

## Abstract

A malformação de Chiari tipo I (MC-I) tem sido associada a distúrbios respiratórios do sono, sobretudo à síndrome de apneia central do sono. Apresentamos o caso de uma paciente do sexo feminino de 44 anos de idade com MC-I que foi encaminhada à nossa unidade de sono por suspeita de apneia do sono. A paciente havia sido submetida a cirurgia descompressiva 3 anos antes. A gasometria arterial mostrou hipercapnia. A polissonografia revelou um índice de distúrbio respiratório de 108 eventos/h, sendo todos os eventos apneias centrais. Foi iniciado tratamento com servoventilação adaptativa e houve resolução da apneia central. Este relato demonstra a eficácia da servoventilação no tratamento da síndrome de apneia central do sono associada à hipoventilação alveolar em uma paciente com MC-I previamente submetida a cirurgia descompressiva.

## Introdução

A malformação de Chiari tipo I (MC-I) é caracterizada pelo deslocamento caudal do cerebelo e pela herniação das amígdalas cerebelares através do forâmen magno.^(^ 1 ^)^ Essa malformação tem sido associada a distúrbios respiratórios do sono, sobretudo à síndrome de apneia central do sono. ^(^ 2 ^)^ O tratamento sintomático da MC-I consiste na descompressão cirúrgica, a qual frequentemente resolve o distúrbio respiratório do sono associado.^(^ 3 ^)^ Relatamos um caso de síndrome de apneia central do sono grave numa doente com MC-I submetida previamente a cirurgia descompressiva, que foi tratada de forma eficaz com servoventilação adaptativa (SVA).

## Relato de caso

Paciente do sexo feminino de 44 anos de idade com diagnóstico de MC-I em 2008 (Figura 1A) foi submetida a cirurgia descompressiva da fossa posterior do crânio. Foram realizadas craniectomia suboccipital, laminectomia C1-C2 e duroplastia. No pós-operatório, registraram-se as seguintes complicações: fístula de liquor, com necessidade de reintervenção com aplicação de cola biológica, e infecção da ferida operatória, que motivou limpeza cirúrgica. Doze meses após a cirurgia, verificou-se um agravamento dos sintomas neurológicos com cefaleias occipitais, vertigens, desequilíbrio na marcha, paresia facial esquerda e disfagia para líquidos. Foi realizada ressonância magnética que mostrou uma malformação óssea na junção craniovertebral associada a uma cavidade siringomiélica de C2 a C3 (Figura 1B). Um novo tratamento cirúrgico não foi efetuado dado o risco associado de depressão respiratória. Três anos após a cirurgia, a paciente foi referenciada à nossa unidade de sono por suspeita de apneia do sono. Referia sono não reparador e cefaleias matinais, mas não apresentava hipersonolência diurna (pontuação de 4 na escala de Epworth), nem sintomas sugestivos de síndrome de pernas inquietas ou de narcolepsia. Segundo informação complementar dos familiares, a paciente apresentava roncopatia e apneias presenciadas. A paciente apresentava história de hipotireoidismo, sendo medicada regularmente com levotiroxina, e não apresentava hábitos tabágicos ou etílicos.

**Figura 1:** Em A, imagem de ressonância magnética cerebral pré-cirurgia demonstrando malformação óssea da junção craniovertebral associada a impressão basilar e clivus encurtado. Amígdalas cerebelares baixas (Malformação de Chiari tipo I), mas sem evidência de siringomielia. Em B, imagem de ressonância magnética cerebral (T2) pós-cirurgia mostrando uma malformação óssea na junção craniovertrebral em associação a siringomielia ao nível de C2 e C3.

Ao exame físico, seu índice de massa corpórea era de 34 kg/m^2^, a pressão arterial sistêmica era de 127/73 mmHg, o perímetro cervical era de 46 cm, apresentando hipertrofia do palato mole (classe II no escore de Mallampati), mas sem dismorfias faciais. O restante do exame era normal.

A radiografia de tórax não mostrou alterações. Não havia evidência de comorbidades cardiovasculares (ecocardiograma e exame de Holter sem alterações). A função da tireoide era normal. A gasometria arterial indicava hipoxemia grave com hipercapnia (FiO_2_ = 0,21; pH = 7,35; PaO_2_ = 51 mmHg; PaCO_2_ = 56 mmHg; e SaO_2_ = 89%). O estudo funcional respiratório revelou uma diminuição leve da CVF com diminuição do volume de reserva expiratória e CPT preservada. A polissonografia mostrou baixa eficiência do sono (44,4%) com 333 eventos respiratórios centrais, um índice de distúrbio respiratório de 108 eventos/h e um tempo de registro com saturação da oxi-hemoglobina < 90% de 27,6% (Figura 2).

**Figura 2:** Registro polissonográfico inicial exibindo apneias centrais.

A paciente iniciou tratamento com SVA com um aparelho Autoset CS S9(tm) (ResMed Corp., San Diego, CA, EUA), com parâmetros de pressão de suporte máxima de 15 cmH_2_O, pressão de suporte mínima de 5 cmH_2_O, pressão expiratória de 8 cmH_2_O e FR de 15 ciclos/min para a síndrome de apneia central do sono. Após seis meses de tratamento, a polissonografia com SVA mostrou uma melhoria do índice de distúrbio respiratório para 4,8 eventos/h e tempo de registro com saturação da oxi-hemoglobina < 90% de 1,4% (Figura 3). Verificou-se também uma melhoria das trocas gasosas (FiO_2_ = 0,21; pH = 7,36; PaO_2_ = 69 mmHg; PaCO_2_ = 46 mmHg; e SaO_2_ = 93%).

**Figura 3:** Registro polissonográfico com o uso de servoventilador mostrando a correção das apneias centrais.

## Discussão

A MC-I tem sido definida como um deslocamento caudal das amígdalas cerebelares acima de 5 mm através do forâmen magno e é geralmente associada a uma fossa posterior volumetricamente reduzida.^(^ 1 ^)^ Esses critérios radiológicos devem ser enquadrados no contexto clínico para o diagnóstico, e a ressonância magnética é o estudo de imagem mais útil. O centro respiratório responsável pelo controle da respiração situa-se no bulbo raquidiano ao nível da transição craniocervical e pode ser afetado nessa patologia, conduzindo assim a distúrbios respiratórios, especialmente durante o sono.^(^ 4 ^)^ Os estudos publicados na literatura têm demonstrado uma elevada prevalência de distúrbios respiratórios do sono em pacientes com MC.^(5,6) ^Na maioria dos casos em adultos, têm sido documentadas sobretudo apneias centrais do sono, mas também têm sido reportadas apneias mistas e obstrutivas.^(^ 7 ^,^ 8 ^)^ As apneias centrais poderiam resultar da compressão direta dos componentes respiratórios centrais, da compressão dos pares de nervos cranianos IX e X e da lesão dos nervos aferentes pelas cavidades siringomiélicas.^(^ 9 ^)^ A hipoventilação é definida como a dessaturação de oxigênio de forma sustentada que não está associada a apneias obstrutivas, hipopneias ou respiração periódica.^(^ 10 ^)^ Os pacientes com hipercapnia diurna, principalmente devido a doença neuromuscular ou anormalidades do controle ventilatório (síndrome de hipoventilação da obesidade e hipoventilação alveolar central), podem apresentar também apneias centrais durante o sono.^(^ 11 ^)^ Os eventos centrais caracterizam-se pela cessação transitória do estímulo neuronal respiratório durante o sono, resultando em diminuição da ventilação e alteração das trocas gasosas.^(^ 12 ^)^ De um modo geral, as apneias centrais durante o sono devem ser distinguidas entre pacientes que apresentam hipercapnia e aqueles que apresentam normocapnia ou hipocapnia. A apneia central do sono hipercápnica sobrepõe-se às síndromes de hipoventilação e é considerada como uma parte integrante da síndrome de hipoventilação do sono.^(^ 11 ^)^

No caso descrito, existiam alterações da função respiratória, caracterizadas por uma redução ligeira da CVF acompanhada da diminuição do volume de reserva expiratória. No paciente obeso há um comprometimento da mecânica respiratória que provoca alterações da função pulmonar, com o aumento do trabalho respiratório e a redução dos volumes pulmonares. A restrição ventilatória associada à obesidade é geralmente leve e é atribuída aos efeitos mecânicos da acumulação de tecido adiposo sobre o diafragma e a parede torácica: a excursão do diafragma é prejudicada e a complacência torácica encontra-se diminuída. ^(^ 13 ^)^ A redução do volume de reserva expiratória pode ser detectável mesmo em pacientes com um aumento modesto de peso. Em casos de obesidade mórbida, essa alteração pode ser acompanhada de uma redução da CPT e da capacidade residual funcional.^(^ 14 ^)^ Alguns pacientes obesos apresentam hipoventilação alveolar. O mecanismo pelo qual a obesidade conduz à hipoventilação é complexo e não se encontra totalmente compreendido. Vários mecanismos têm sido propostos, incluindo alteração da mecânica ventilatória, respostas centrais diminuídas para hipercapnia e hipóxia e alterações neuro-hormonais, como resistência à leptina.^(^ 15 ^)^ A síndrome de obesidade/hipoventilação é definida como uma combinação de obesidade (índice de massa corpórea ≥ 30 kg/m^2)^, hipercapnia diurna e vários tipos de distúrbios respiratórios do sono após a exclusão de outras patologias que podem originar hipoventilação alveolar (doenças pulmonares obstrutivas ou restritivas, patologias da parede torácica e doenças neuromusculares).^(^ 16 ^)^ Pacientes com distúrbios neurológicos, incluindo a MC, podem apresentar hipoventilação central. ^(^ 17 ^)^ Contudo, a hipoventilação alveolar associada a apneias centrais não é frequente na MC-I, e, no presente caso, não se pode excluir o papel da obesidade nas alterações observadas na gasometria arterial.

A cirurgia descompressiva habitualmente resulta na diminuição dos eventos respiratórios durante o sono e reduz a fragmentação do sono em um número significativo de pacientes, efeitos mais pronunciados naqueles com apneias centrais. ^(^ 3 ^)^ No entanto, há relatos de aparecimento de apneias centrais após cirurgia.^(^ 18 ^)^

A SVA é um modo ventilatório controlado por pressão e em ciclos por volume ou pico de fluxo. A SVA corrige as apneias centrais, fornecendo um ajuste dinâmico (respiração por respiração) da pressão de suporte inspiratória com uma frequência respiratória de *backup* para normalizar o padrão respiratório. A eficácia da SVA tem sido demonstrada sobretudo no tratamento da síndrome de apneia central do sono associada a insuficiência cardíaca congestiva. Na síndrome de apneia central do sono associada a distúrbios neurológicos (sem respiração periódica do tipo Cheyne-Stokes), o papel da SVA não se encontra ainda bem estabelecido.^(^ 19 ^)^

O caso clínico demonstra a eficácia da SVA no tratamento da síndrome de apneia central do sono associada a hipoventilação alveolar em uma paciente com MC-I, pois observou-se uma completa resolução dos eventos centrais e uma melhoria significativa das trocas gasosas. Além disso, o caso sugere que a SVA pode ser eficaz no tratamento de apneia central do sono associada a MC-I após a descompressão cirúrgica. Na literatura publicada, foi encontrada apenas uma referência a um caso clínico idêntico.^(^ 20 ^)^ Desse modo, a SVA pode ser uma alternativa à cirurgia descompressiva no tratamento da apneia central do sono associada a MC-I.
